# InterPro in 2017—beyond protein family and domain annotations

**DOI:** 10.1093/nar/gkw1107

**Published:** 2016-11-28

**Authors:** Robert D. Finn, Teresa K. Attwood, Patricia C. Babbitt, Alex Bateman, Peer Bork, Alan J. Bridge, Hsin-Yu Chang, Zsuzsanna Dosztányi, Sara El-Gebali, Matthew Fraser, Julian Gough, David Haft, Gemma L. Holliday, Hongzhan Huang, Xiaosong Huang, Ivica Letunic, Rodrigo Lopez, Shennan Lu, Aron Marchler-Bauer, Huaiyu Mi, Jaina Mistry, Darren A. Natale, Marco Necci, Gift Nuka, Christine A. Orengo, Youngmi Park, Sebastien Pesseat, Damiano Piovesan, Simon C. Potter, Neil D. Rawlings, Nicole Redaschi, Lorna Richardson, Catherine Rivoire, Amaia Sangrador-Vegas, Christian Sigrist, Ian Sillitoe, Ben Smithers, Silvano Squizzato, Granger Sutton, Narmada Thanki, Paul D Thomas, Silvio C. E. Tosatto, Cathy H. Wu, Ioannis Xenarios, Lai-Su Yeh, Siew-Yit Young, Alex L. Mitchell

**Affiliations:** 1 European Molecular Biology Laboratory, European Bioinformatics Institute (EMBL-EBI), Wellcome Trust Genome Campus, Hinxton, Cambridge CB10 1SD, UK; 2 School of Computer Science, University of Manchester, UK; 3 Department of Bioengineering & Therapeutic Sciences, University of California, San Francisco, CA 94143, USA; 4 European Molecular Biology Laboratory, Biocomputing, Meyerhofstasse 1, 69117 Heidelberg, Germany; 5 Swiss-Prot Group, SIB Swiss Institute of Bioinformatics, CMU, 1 rue Michel-Servet, CH-1211 Geneva 4, Switzerland; 6 MTA-ELTE Lendület Bioinformatics Research Group, Department of Biochemistry, Eötvös Loránd University, Pázmány Péter sétány 1/c, Budapest, Hungary; 7 Computer Science department, University of Bristol, Woodland Road, Bristol BS8 1UB, UK; 8 Bioinformatics Department, J. Craig Venter Institute, 9714 Medical Center Drive, Rockville, MD 20850, USA; 9 Center for Bioinformatics and Computational Biology, University of Delaware, Newark, DE 19711, USA; 10 Division of Bioinformatics, Department of Preventive Medicine, University of Southern California, Los Angeles, CA 90033, USA; 11 Biobyte Solutions GmbH, Bothestr. 142, 69126 Heidelberg, Germany; 12 National Center for Biotechnology Information, National Library of Medicine, NIH Bldg, 38A, 8600 Rockville Pike, Bethesda, MD 20894, USA; 13 Georgetown University Medical Center, 3300 Whitehaven St, NW, Washington, DC 20007, USA; 14 Department of Biomedical Sciences and CRIBI Biotech Center, University of Padua, via U. Bassi 58/b, 35131 Padua, Italy; 15 Structural and Molecular Biology, University College London, Darwin Building, London WC1E 6BT, UK; 16 CNR Institute of Neuroscience, via U. Bassi 58/b, 35131 Padua, Italy

## Abstract

InterPro (http://www.ebi.ac.uk/interpro/) is a freely available database used to classify protein sequences into families and to predict the presence of important domains and sites. InterProScan is the underlying software that allows both protein and nucleic acid sequences to be searched against InterPro's predictive models, which are provided by its member databases. Here, we report recent developments with InterPro and its associated software, including the addition of two new databases (SFLD and CDD), and the functionality to include residue-level annotation and prediction of intrinsic disorder. These developments enrich the annotations provided by InterPro, increase the overall number of residues annotated and allow more specific functional inferences.

## INTRODUCTION

In the post-genomic era, generation of biological sequence data is no longer a scientific barrier; rather, data storage and analysis have become the new bottlenecks in terms of cost and time (1). With the potential to sequence entire genomes, or to generate hundreds of millions of sequences from environmental samples, the pace of generating sequence data now outstrips the rate of experimental characterisation by many orders of magnitude (2). Consequently, rapid, accurate automatic functional annotation of large numbers of sequences has become a major challenge.

The InterPro database aims to meet this challenge by integrating diverse information about protein families, domains and functional sites. Central to the resource are diagnostic models (profile hidden Markov models (HMMs), profiles, position-specific scoring matrices or regular expressions, collectively known as ‘signatures’), against which protein sequences can be searched to determine their potential functions. The signatures are provided by 14 different member databases: 12 of these are long-standing members of the InterPro Consortium (CATH-Gene3D (3), HAMAP (4), PANTHER (5), Pfam (6), PIRSF (7), PRINTS (8), ProDom (9), PROSITE Patterns (10), PROSITE Profiles (10), SMART (11), SUPERFAMILY (12) and TIGRFAMs (13)); two are new members, the Conserved Domains Database (CDD) (14) and Structure–Function Linkage Database (SFLD) (15) having been added in 2016.

The source databases each have their own individual biological focus, method of signature production, and/or signature-match processing. The diversity of approaches helps to ensure that annotations are as comprehensive as possible. For example, related Pfam (profile HMM-based) and Prosite Profiles entries often match subtly different sets of proteins; united however, they match most, if not all, known members of a protein family, while eliminating false-positive annotations. Furthermore, the different databases offer complementary levels of protein classification, from broad-level (*e.g*., a protein is a member of a superfamily) to more fine-grained assignments (e.g. a protein is a member of a specific family, or possesses a particular type of domain). These different levels of granularity are used by InterPro to produce a hierarchical classification system: one or more-member database signatures are integrated into an InterPro entry, and, where appropriate, relationships are highlighted between different entries, identifying those that represent smaller, functionally specific subsets of a broader entry.

### Database curation

InterPro entries are classified into types (families, domains, repeats or sites) depending on the biological entity they represent. Family and domain entries are placed into distinct, non-overlapping hierarchies: domain entries are able to occur in the same hierarchy as other domains, but not within the same hierarchy as family entries, and vice versa.

**Figure 1.**
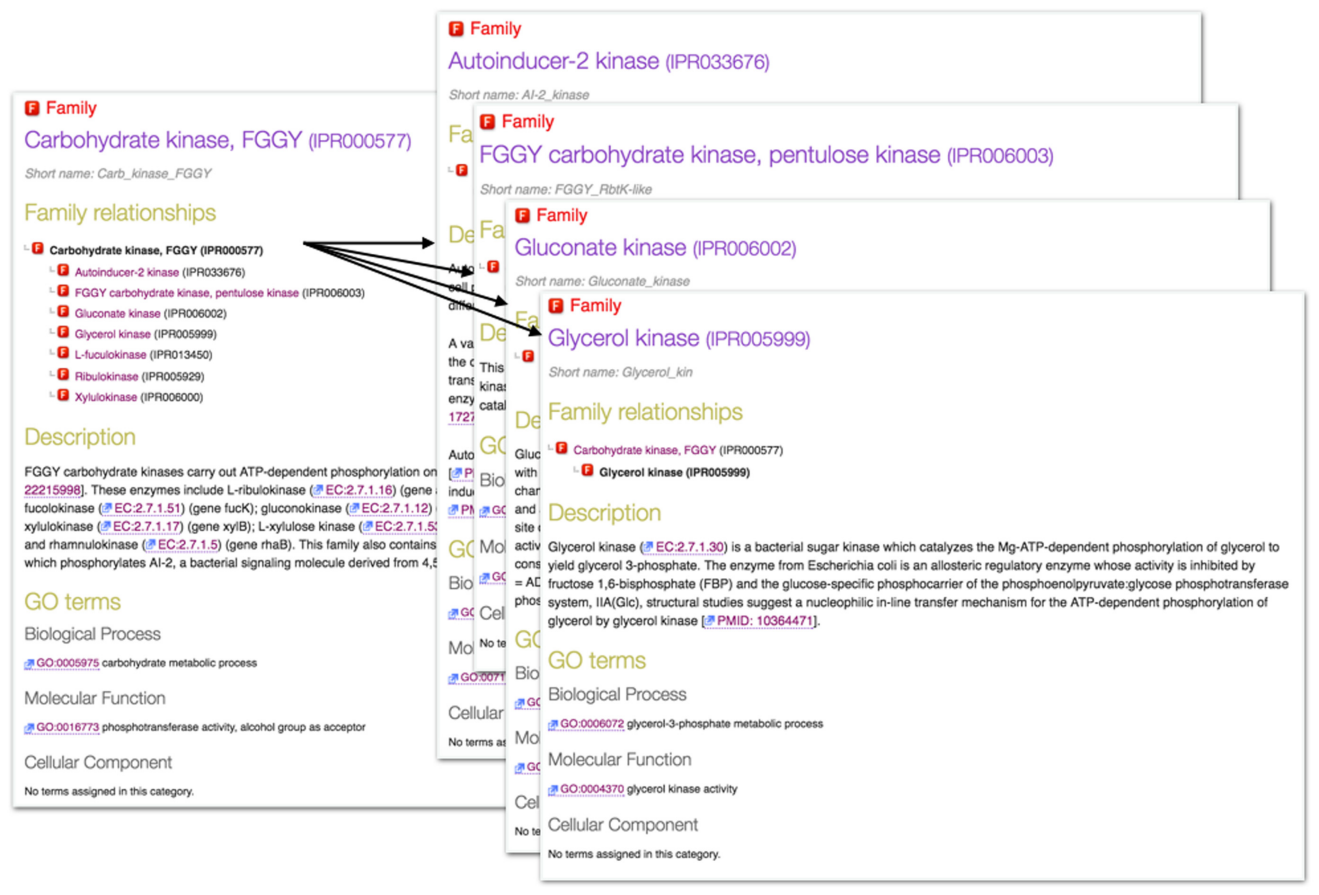
Example of an InterPro family hierarchical relationship. The FGGY carbohydrate kinases entry (IPR000577) provides a parent to a series of child entries that match smaller, more functionally-specific sets of proteins.

Entries are manually annotated with literature-referenced free-text descriptions, explaining the biological information that may be inferred for proteins that match a given signature. Where possible, each entry is also associated with Gene Ontology (GO) (16) terms, which provide a controlled vocabulary to describe protein function, cellular localisation and involvement in wider biological pathways and processes. The granularity of the member database signatures (and hence InterPro entries) determines the specificity of the functional annotation and GO terms that can be assigned. For example, an InterPro entry representing a small family of functionally conserved enzymes that act on a single substrate, such as the glycerol kinases (IPR005999), is annotated with more specific functional information and terms from the GO hierarchy than an entry representing a more diverse enzyme family acting on a larger class of substrates, such as their parent InterPro entry, the FGGY carbohydrate kinases (IPR000577) (see Figure 1).

At each release of the database, InterPro entries are checked, and updated where necessary, to ensure that the annotations remain accurate. Updates to annotations are typically made in response to changes in signature specificity (e.g. if a signature has been rebuilt by a member database to recognise a tighter functionally-related group of proteins, or to match more distant homologues) or to improved scientific understanding of the function of a protein family (17).

InterPro entries are also automatically annotated with cross-references to a range of relevant databases, including the pathway databases ENZYME (18), MetaCyc (19), UniPathway (20) and KEGG (21), and various 3D structure databases (22,23).

### Use of the resource

InterPro plays a major role in the analysis and annotation of sequence data held in the UniProt Knowledgebase (UniProtKB), the central hub of protein sequences (24). Protein annotations derived from InterPro's member database signatures are calculated using the InterProScan software package (25) on a monthly basis. These annotations are then used by UniProtKB curators to help annotate Swiss-Prot records and as input to the automated systems that add annotation to UniProtKB/TrEMBL. InterPro's protein match information is also made available to the public via XML files, and the database's Web interfaces and services, which can be searched with a protein sequence, a UniProtKB protein identifier, an InterPro or member database identifier, GO term, or free text.

In addition to its use in UniProtKB annotation, InterPro is widely used by the scientific community. Its data feed into a host of annotation pipelines, including ENSEMBL (26), ENSEMBL Genomes (27), PDBe (28), BLAST2GO (29), PhytoPath (30), the digenic diseases database - DIDA (31), and the Endeavour candidate gene prioritisation server (32). The InterProScan Web services are extensively used, processing in excess of 40 million sequence searches per month. InterPro's data and analysis software are increasingly used in the analysis of metagenomic data: in addition to underpinning EMBL-EBI's in-house EBI Metagenomics resource (33), the MEGAN metagenomics analysis tool (34) now has the ability to process InterPro-derived data and GO terms.

### New protein family member databases

**Figure 2.**
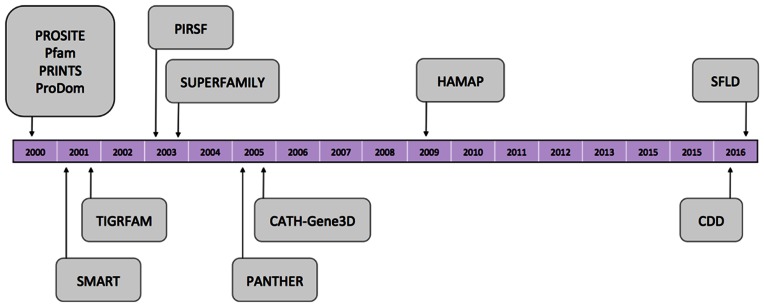
Timeline showing the member databases that have joined InterPro since version 1.0, released in 2000.

The constituent databases of InterPro have remained fixed for the last 7 years (see Figure 2), and have changed little in the last decade. Since the last update paper (35), we have been evaluating a range of different databases that could enhance the information contained within InterPro, in terms both of adding more comprehensive coverage and in providing more fine-grained functional annotations at the individual amino acid level. As a result, two new databases have been added to the resource: the Conserved Domains Database (CDD) and Structure–Function Linkage Database (SFLD).

CDD is a manually curated protein annotation resource representing domain footprints conserved in molecular evolution. Each domain entry is modelled as a multiple sequence alignment, which is also converted into a position-specific scoring matrix (PSSM) that allows fast identification of conserved domains in protein sequences via RPS-BLAST (36). NCBI-curated domains use 3-dimensional structure information to explicitly define the boundaries of known conserved domains, and to provide comprehensive and accurate annotation of protein sequences with the locations and boundaries (footprints) of known conserved domains, including location of conserved/functional sites. CDD content also includes domain models imported from a number of external source databases (Pfam, SMART, COG (37), PRK (38) and TIGRFAMs). Only CDD's own models have been imported into InterPro, mainly owing to the fact that Pfam, SMART and TIGRFAMs are already present in InterPro. Within InterProScan, the RPS-BLAST is substituted by a piece of software called ‘rpsbproc’, an amendment to the standalone RPS-BLAST program, which allows detailed CD-Search results, including domain superfamily assignments and the predicted locations of conserved sites to be reproduced locally. As well as being bundled into InterProScan, the rpsbproc utility is available from the CDD FTP site at ftp://ftp.ncbi.nih.gov/pub/mmdb/cdd. This package ensures that InterPro can faithfully reproduce the results from CDD, thus ensuring consistency of annotations between the CDD and InterPro Web servers for the same sequence.

SFLD is a manually-curated classification resource describing structure-function relationships for functionally diverse enzyme superfamilies. Members within a single superfamily are derived from a common ancestor and mediate a diverse set of related, yet distinct reactions. For example, the enzymes within a superfamily may share active-site features (such as residues in a nucleophile) associated with conserved functional attributes (e.g. part of a reaction mechanism or substrate binding). Consequently, in some annotation resources, such superfamilies are grouped together in a single entry (e.g. the Radical_SAM entry in Pfam, PF04055), but are inadequately subdivided into their functional groups, leading to imperfect annotations.

**Figure 3.**
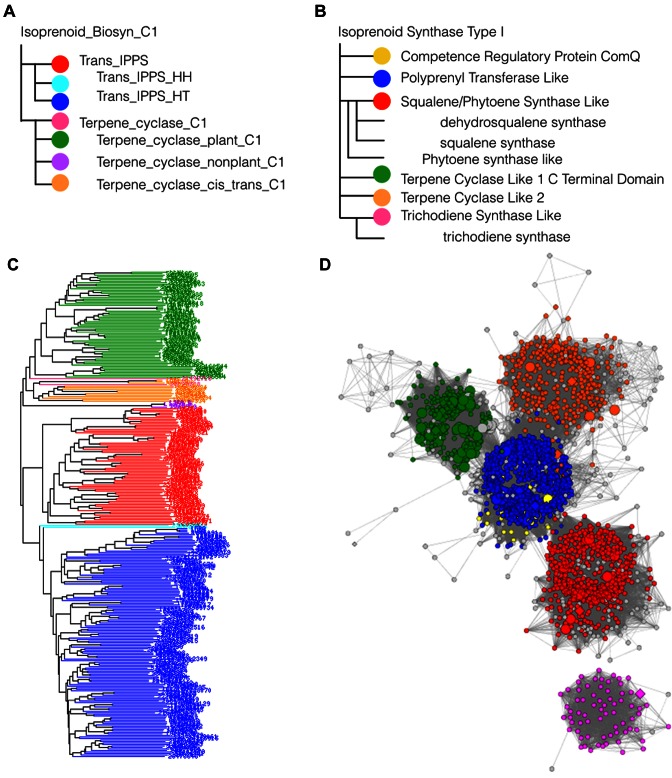
Examples of the CDD and SFLD hierarchies (A and B). (**A**) CDD models for related domains are organized hierarchically, reflecting major events in the domain family's molecular evolution and functional diversification. The hierarchy usually follows a tree structure obtained from (**C**) phylogenetic analysis of multiply aligned sequences. The relationship between the CDD entries in panel A and the sequences in panel B is indicated by colour. The top ‘parent’ entry (isoprenoid biosynthesis enzymes, Class 1 superfamily) would be less specific than the ‘leaf’ node entry (trans-isoprenyl diphosphate synthase, head-to-head). (**B**) The corresponding superfamily, Isoprenoid Synthase Type I, from SFLD. The specificity relationships between the entries is similarly arranged as in panel A. (**D**) SFLD network analysis graph showing the sequence identity relationships between the Isoprenoid Synthase Type I superfamily members. The *E*-value threshold for the network is 1e-10 and sequences within nodes share 50% or more sequence identity, calculated using CD-HIT. Note, figures C and D are visualizations from the respective source database and are not available from the InterPro website. These figures demonstrate the different approaches for visualizing and defining relationships between families.

To overcome annotation errors and enable transfer of functional features, SFLD provides hierarchical annotations at multiple levels. SFLD subdivides superfamilies into subgroups based upon sequence information, then into families of enzymes known to catalyze the same reaction using the same mechanistic strategy. The family level of the hierarchy defines variations in each set of active site residues that distinguish that family's particular reaction type from other families in the superfamily (Figure 3).

To enable InterPro to perform SFLD annotations, the two resources have developed an approach whereby SFLD produces multiple sequence alignments representing the different superfamilies, subgroups and families. Most of the alignments, particularly those associated with subgroups and families, are annotated with key catalytic residues that are important to the chemical reaction performed by the sequences found within that set. From these alignments, InterPro builds a profile HMM library that is used to annotate sequences. Significant sequence matches are then verified against the key catalytic residues (if present), only those sequences matching all residues being assigned to the family. Thus, the profile HMMs and residue verification steps act as a two-stage assignment criterion, enabling very fine-grained annotations to be made, at rates only marginally slower than searching profiles alone.

### Integration plans for CDD and SFLD

When a new member database is added to InterPro, the database is added as an entire set of unintegrated signatures, which are then manually annotated and added to InterPro entries, as described above, by the curation team. Thus, CDD and SFLD matches are now available for all signatures, but require a large curation effort to fully integrate each new resource within the InterPro hierarchy. Until integrated, the annotations from these databases are provided at the bottom of each protein page, in the ‘Detailed signature matches’, under the ‘unintegrated signatures’ listing. They are provided in a similar way in the InterProScan output. For each member database, it is possible to get the complete listing of signatures from the following pages: http://www.ebi.ac.uk/interpro/member-database/<member-database>, for example http://www.ebi.ac.uk/interpro/member-database/CDD for the CDD database.

As of InterPro release 58.0, CDD version 3.14 added 11 273 signatures, of which 1,005 have now been integrated into InterPro over a period of 4 months. During this time, there has been a dual approach to integrating CDD signatures: (i) adding those that are directly equivalent to other member database signatures that have already been included in InterPro, as this can be done with minimal curation; (ii) adding those that match sequence sets not covered by any other member database, as these add coverage to InterPro.

SFLD, a smaller scale database with a more specific focus, has been added to InterPro more recently. SFLD version 1.0 provided 480 signatures in total, 17 of which were integrated over a 2-week period into the most recent InterPro 60.0 release. Increasing the number of integrated signatures from these databases will be a significant focus for InterPro in the forthcoming year.

Both CDD and SFLD provide hierarchical classifications (see Figure 3), but their hierarchies differ from each other and from that of InterPro. In the case of CDD, the parent signature in the hierarchy does not aim to match all of the sequences matched by its children. Rather, CDD parent entries often provide signatures aiming to cover the relatively few family member sequences that are not matched by the child signatures. As such, one of the most important functions of the parent entry is to provide a root node through which child signatures can be associated. This is significantly different from the hierarchical classification approach used in InterPro, where a parent level entry should (if possible) provide coverage of all proteins matching its child entries. As a result, CDD's higher-level signatures will not be integrated into InterPro. Instead, the aim will be to identify signatures from other member databases that can be substituted in their place, grouping the child entries together.

The SFLD hierarchy is closer to that provided by InterPro, but with some important differences. Superfamily-level entries in SFLD tend to be based upon the common amino acid core of a set of matching sequences. Descending the hierarchy, the child signatures tend to increase in length, pulling in more accessory domains, which provide functional specificity. As a result, while most SFLD entries correspond to InterPro family-type entries, some superfamilies may appear more like domains (for example, where the functional unit of a sequence family is represented only by a single domain). InterPro aims to follow the SFLD hierarchy as closely as possible, integrating SFLD entries as ‘type’ Family. Nevertheless, some discrepancies will inevitably occur as we try to merge this hierarchy with InterPro's hierarchy. This is consistent with the current integration strategy for member databases that define families and domains slightly differently from InterPro.

### Updates to data content

InterPro is released publicly every 2 months, with a break in production during August, in harmony with the UniProtKB release cycle. With each InterPro release, one or more of its member databases may have been updated, providing a stream of new entries for integration into the resource. There have been 12 public InterPro releases since the last update paper, with an additional 5,158 signatures being integrated into 3,462 new InterPro entries: 4,035 of these signatures came from existing member databases, and a further 1123 from CDD and SFLD. The latest release (version 60.0) contains 41 925 member database signatures integrated into 29 700 InterPro entries. The member database updates that contributed to recent InterPro releases are shown in Table 1.

**Table 1.** Member database release versions integrated into InterPro since release 48.0

InterPro release	Member database update
49.0	PROSITE patterns (20.105), PROSITE profiles (20.105)
50.0	PIRSF (3.01)
51.0	TIGRFAMs (15.0), HAMAP (201502.04)
52.0	PROSITE patterns (20.113), PROSITE profiles (20.113)
53.0	Pfam (28.0)
54.0	PANTHER (10.0)
55.0	HAMAP (201511.02)
56.0	PROSITE patterns (20.119), PROSITE profiles (20.119)
57.0	Pfam (29.0), SMART (7.1)
58.0	CDD (1.0)*, HAMAP (201605.11)
59.0	Pfam (30.0), SFLD (1.0)*
60.0	MobiDB**

*New member databases.

**MobiDB is a new non-signature based database that has been integrated into InterPro to provide ID region annotations. See text for details.

The InterPro coverage of sequences in UniProtKB (i.e. the number of proteins with one or more InterPro annotations) is calculated at each release. The signatures integrated into InterPro 60.0 provide matches to 79.8% of the sequences in UniProtKB release 2016_09 (see Table 2), compared to 83.5% in release 48.0. GO terms assigned by the InterPro2GO pipeline (which associates terms with proteins based on their InterPro matches) are cross-referenced more than 130 million times in UniProt 2016_09, representing annotation for 42 million individual proteins. This compares to 168 million terms for almost 50 million proteins in InterPro release 48.0/UniProtKB 2014_07.

**Table 2.** Coverage of the major sequence databases UniProtKB and UniParc (the non-redundant protein sequence archive) by InterPro signatures

Sequence database	Number of proteins in database	Number of proteins with one or more matches to InterPro
UniProtKB/Swiss-Prot	552 884	533 303 (96.5%)
UniProtKB/TrEMBL	70 656 157	56 310 112 (79.7%)
UniProtKB (total)	71 209 041	56 843 415 (79.8%)
UniParc	132 489 873	103 835 823 (78.4%)

The reduction in InterPro's coverage of UniProtKB since our last report may seem counterintuitive, especially as the number of sequences in UniProtKB has decreased (from a peak of 90 million sequences during 2015, to the current level of 71 million), while the number of InterPro entries and associated GO terms has gone up (the latter increasing from ∼28 000 in release 48.0 to >32 000 in the current release). However, the reduction in the number of records in UniProtKB has been brought about as a redundancy removal effort, aimed at eliminating close to identical proteomes that are over-represented in the database. As part of this process, UniProtKB records belonging to ∼15 000 redundant bacterial proteomes were moved from UniProtKB into UniParc. As InterPro provided high levels of coverage for these proteomes, their removal meant that InterPro's overall coverage of UniProtKB has been disproportionately affected. Increasing coverage of UniProtKB's smaller, but more diverse, sequence set will be an ongoing challenge for InterPro and its member databases in the coming years.

### Per residue annotations

We have been investigating ways to expand the scope of InterPro annotations; specifically, to individual residues that fall within a region defined by a signature. As signature-derived matches are currently based on scores across the entire matched region, the methods can often fail to discriminate between functionally distinct groups. For example, the InterPro entry for the calpain catalytic domain (IPR001300) matches >6000 UniProtKB sequences. While these are undoubtedly derived from a common ancestor, in ∼2500 cases the active site residues have been mutated to residues that are no longer capable of performing the proteolytic reaction. Thus, many of the sequences are not active peptidases, but are likely to perform different functions: e.g. calpamodulin (also known as calpain 6) has the catalytic Cys replaced by Lys, and is a microtubule-stabilising protein, particularly in embryonic muscle, where it has been shown to suppress skeletal muscle differentiation (39). One approach to achieve discrimination between active and inactive forms is to have specific subfamily signatures, e.g. the PANTHER subfamily model for calapin-6: PTHR10183:SF355. However, this requires the production and curation of many models, and there will always be cases where it is hard or impossible to ensure that the signature is capable of separating active and inactive forms, or active forms with (subtly) different mechanisms of action. In such cases, accurately annotating active site residues would help separate active peptidases from inactive homologues.

The mechanism described above is exactly the one employed by the SFLD database to identify specific groups of proteins within entries. Thus, the integration of SFLD into InterPro brings not only increased protein family coverage, but also the annotation of thousands of important residues. Other InterPro databases also provide residue-level annotations: CDD's RPS-BLAST matrices are annotated with a range of per-residue annotations, including active sites, ligand-binding, protein-protein interactions and nucleic acid-protein interactions; Pfam contains active site annotations, based on matches to UniProtKB/Swiss-Prot sequences; PIRSF annotations can also be extended to residues, using the PIRSR resource; and HAMAP and PROSITE annotation rules provide external users and the UniProt automatic annotation pipeline (UniRule) with annotations for functionally important residues (single residues as well as continuous and discontinuous motifs).

To enable the capture of this fine-grained information, we have extended the InterPro data model to deal with per-residue annotations. To date, these annotations have been enabled in InterProScan for SFLD and CDD, with the aim of adding other databases that also provide per-residue annotations in future releases. These developments provide a further tier to the annotations already provided by InterPro. They will also underpin future opportunities to improve annotation granularity. Specifically, the data will allow families to be subdivided into more fine-grained functional groups based on residue patterns, will allow specific annotations to be provided for entries (e.g. identifying the critical functional residues for a given catalytic domain), and will enable the adoption of rule-based approaches (similar to those used by HAMAP, PIRSR and PROSITE) for the assignment of specific functional annotations, such as GO terms.

### Other sequence features annotated by InterPro

For a number of years, InterProScan has included the capability to annotate signal peptides, transmembrane regions and coiled-coils, drawing upon a suite of algorithms to make these annotations (specifically, Coils v2.2, Phobius v1.01, SignalP v4.1 and TMHMM v2.0 in the latest InterProScan release). To complement these, we have integrated a new tool called MobiDB Lite, which provides a consensus prediction of long disordered regions.

**Figure 4.**
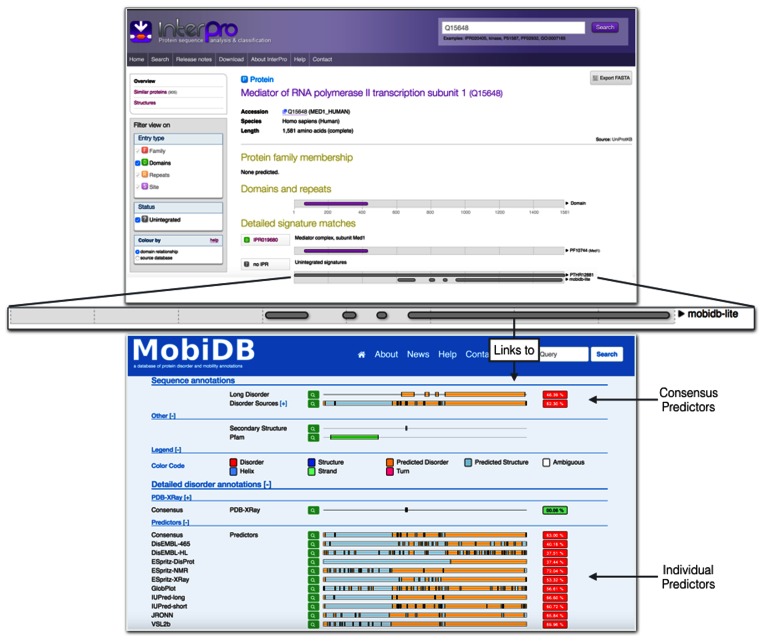
Integration of MobiDB Lite annotation within InterPro, enabling annotation of intrinsic disordered (ID) regions within proteins. **Top** - InterPro annotations for the Human mediator of RNA polymerase II transcription subunit 1 protein (UniProtKB accession Q15648). **Middle** - Zoomed in view of the consensus long range ID predictions provided by MobiDB Lite. InterPro only captures the consensus output for each sequence, but the graphical representations of the ID regions link to the source website, MobiDB (**bottom)**, where the individual predictions can be viewed.

Intrinsically disordered (ID) protein regions, which do not adopt a single well-defined conformation in isolation, rely on a highly flexible state or structural plasticity to carry out their functions (40,41). While ID regions are present in all three domains of cellular life, they often exhibit very little evolutionary conservation and are hence difficult to predict using signature based methods currently employed by InterPro member databases. Information about ID regions largely complements domain and family annotations. At a closer look, ID encompasses different phenomena and different predictors can capture complementary aspects (42,43). MobiDB Lite combines different predictors to generate a consensus focusing on long disordered regions. Eight different algorithms (IUPred-short, IUPred-long (44), GlobPlot (45), DisEMBL-465, DisEMBL-HL (46), Espritz-DisProt, Espritz-NMR and Espritz-X-ray (47)) were chosen for their speed and orthogonality of approaches. The consensus is generated by evaluating the agreement among predictors and smoothing short disorder stretches. A strict agreement threshold of at least 5 out of 8 methods favors precision over inclusiveness and a length cutoff (≥20 residues) helps to discriminate functional disorder from ambiguous assignments. MobiDB lite annotations have been enabled in InterProScan, and are available to external users in the InterProScan 5.20–60.0 release, part of InterPro release 60.0. Graphical representations of ID are also to be implemented on InterPro's Web interfaces (see Figure 4). From each ID region, InterPro provides a link to the MobiDB (43) page for the protein of interest where the breakdown of the individual predictions and additional ID annotation may be found. The MobiDB Lite consensus corresponds to the ‘Long Disorder’ track in MobiDB.

## DISCUSSION

Since its inception 17 years ago, InterPro has striven to provide a comprehensive protein classification resource that enables high-quality functional annotation of protein sequences. It has met this aim in collaboration with its member databases, which have provided an invaluable stream of signatures for integration into the resource. As a result, InterPro has grown significantly in terms of coverage and function-annotation specificity, and has developed a substantial worldwide user-base. During this time, most of the member databases have evolved to adopt new algorithms and include new data. For example, eight of the nine profile HMM-based member database now use the significantly faster version of HMMER, version 3.0 (with only SMART using HMMER 2.0). Meanwhile, for calculating sequence matches to HAMAP within InterProScan, the two teams developed a heuristic (using a profile HMM trained on the same alignment used to produce the HAMAP profile and used as pre-filter search), to ensure continued scalability. As indicated in Table 1, 10 of the 14 member databases (CDD, HAMAP, PANTHER, Pfam, PIRSF, Prosite Patterns, Prosite Profiles, SFLD, SMART, TIGRFAM) have been added or updated at least once over the past two years, with CATH-Gene3D awaiting update from version 3.5 to 4.1 in InterPro, demonstrating a steady increase of new data and sustained curation effort from InterPro's member databases. Two new member databases CDD and SFLD further increase coverage and extend the number of resources that annotate discrete functional amino acid residues; this functionality is now available in InterPro for the first time. We will continue to work with our expert member databases, to provide more per-residue annotations, e.g. HAMAP and PROSITE both define annotations dependent on features composed of multiple non-contiguous residues.

This additional tier of annotations is a step change for InterPro: it adds a feature that has long been absent from the resource, and, alongside the intrinsic disorder predictions from MobiDB Lite (to complement membrane-topology and coiled-coil prediction), enables the most richly-detailed, informative annotation of protein sequences possible. The integration of similar residue-level annotations from databases like Pfam and PIRSF, coupled with highly specific subfamily-level annotations from resources like PANTHER and PRINTS, extends this functionality even further. Together, these advances will help improve the annotation of proteins in databases like UniProtKB, by adding more discriminatory power to automated annotation systems like UniRule. InterPro's Web interfaces will be expanded to present this additional layer of annotation to users, ensuring both added value and continued usefulness of the resource for the scientific community.
